# Tracking Economic Value of Products in Natural Settings: A Wireless EEG Study

**DOI:** 10.3389/fnins.2018.00910

**Published:** 2018-12-20

**Authors:** Hannah Roberts, Vicente Soto, John Tyson-Carr, Katerina Kokmotou, Stephanie Cook, Nicholas Fallon, Timo Giesbrecht, Andrej Stancak

**Affiliations:** ^1^Department of Psychological Sciences, Institute of Psychology, Health and Society, University of Liverpool, Liverpool, United Kingdom; ^2^Institute for Risk and Uncertainty, University of Liverpool, Liverpool, United Kingdom; ^3^Division of Psychology, De Montfort University, Leicester, United Kingdom; ^4^Unilever Research & Development, Port Sunlight, United Kingdom

**Keywords:** BDM, eye-movement related potentials, value, source dipole analysis, mobile EEG

## Abstract

Economic decision making refers to the process of individuals translating their preference into subjective value (SV). Little is known about the dynamics of the neural processes that underpin this form of value-based decision making and no studies have investigated these processes outside of controlled laboratory settings. The current study investigated the spatio-temporal dynamics that accompany economic valuation of products using mobile electroencephalography (EEG) and eye tracking techniques. Participants viewed and rated images of household products in a gallery setting while EEG and eye tracking data were collected wirelessly. A Becker-DeGroot-Marschak (BDM) auction task was subsequently used to quantify the individual’s willingness to pay (WTP) for each product. WTP was used to classify products into low, low medium, high medium and high economic value conditions. Eye movement related potentials (EMRP) were examined, and independent component analysis (ICA) was used to separate sources of activity from grand averaged EEG data. Four independent components (ICs) of EMRPs were modulated by WTP (i.e., SV) in the latency range of 150–250 ms. Of the four value-sensitive ICs, one IC displayed enhanced amplitude for all value conditions excluding low value, and another IC presented enhanced amplitude for low value products only. The remaining two value-sensitive ICs resolved inter-mediate levels of SV. Our study quantified, for the first time, the neural processes involved in economic value based decisions in a natural setting. Results suggest that multiple spatio-temporal brain activation patterns mediate the attention and aversion of products which could reflect an early valuation system. The EMRP parietal P200 component could reflect an attention allocation mechanism that separates the lowest-value products (IC7) from products of all other value (IC4), suggesting that low-value items are categorized early on as being aversive. While none of the ICs showed linear amplitude changes that parallel SV’s of products, results suggest that a combination of multiple components may sub-serve a fine-grained resolution of the SV of products.

## Introduction

Rewarding and aversive stimuli that occur in natural environments prompt humans to make a large number of value based decisions. Such decisions can be computed consciously, or can be reached without conscious awareness. Determining the subjective value (SV) of each parallel option is a key input for the decision process. Economic decisions occur when an individual evaluates how much a product is worth by computing subjective preferences reflecting their current needs and desires (Gluth et al., 2012; Polanía et al., 2014; Ruff and Fehr, 2014; Stott and Redish, 2015). According to the value maximization framework (Samuelson, 1937; Kahneman and Tversky, 1979), economic decisions involve the initial assignment of SVs to competing alternatives and the option with the highest SV is consequently selected (Rangel et al., 2008; Wallis and Rich, 2011; Bartra et al., 2013).

There is only a limited number of studies examining the temporal sequencing of economic value-based decisions in the brain using event related potential (ERP) methods, particularly for low value products (Gajewski et al., 2016; Goto et al., 2017). Although limited, some studies have suggested that the N200 visually evoked potential (VEP) represents an early selective attentional response to relevant stimuli and, hence, could be related to consumer preferences (Telpaz et al., 2015; Goto et al., 2017; Tyson-Carr et al., 2018). For example, Telpaz et al. (2015) employed a binary choice paradigm and found a smaller N200 for preferred products. Likewise, the P200, which is thought to index early selective attention, has also been implicated in economic decision making and buying decisions (Jones et al., 2012; Pozharliev et al., 2015; Tyson-Carr et al., 2018). Studies regarding later ERP components in relation to economic decisions, however, show conflicting results (Pozharliev et al., 2015; Telpaz et al., 2015; Goto et al., 2017). For instance, Pozharliev et al. (2015) found that the late positive potential (LPP) was modulated by product preferences for luxury items, but only when in the presence of others. These components detailed above, including the N200 (Handy et al., 2010), P200 (Carretié et al., 2001b), and LPP (Schupp et al., 2000), have also been implicated in general hedonic processing. An important question for researchers is how making economic value-based decisions for low value items differs from high value items, and whether such decisions employ the same neural circuitry (Xie and Padoa-Schioppa, 2016) or multiple neural systems (Dickinson and Balleine, 2002; Daw et al., 2005; Rangel et al., 2008).

The SV of a good can be approximated by the amount of money an individual is willing to pay for an item (Chib et al., 2009; Peters and Büchel, 2010). WTP can be estimated using the BDM auction (Becker et al., 1964). The construction of the auction is such that a value smaller or larger than the actual value that a participant would be willing to pay would produce a disadvantageous outcome, whereas bidding their true WTP would maximise expected utility (Berry et al., 2012). The validity of the BDM has been supported by experiments demonstrating that it reliably activates brain areas that have been associated with value processing, such as the medial orbitofrontal cortex (Plassmann et al., 2007, 2010), the ventral striatum (De Martino et al., 2009) and the dorsomedial prefrontal cortex (Plassmann et al., 2007). The BDM auction paradigm is motivationally relevant as the subject receives a subset of items that have been purchased successfully, making it more likely that subjects will provide a true economic value (Keller et al., 1993). Recently, Tyson-Carr et al. (2018) used the BDM auction to examine the spatio-temporal dynamics of economic decisions for everyday household products in a laboratory-based setting. A negative component peaking at about 200 ms with a source in the right anterior insula was found to be stronger in low compared to high value products, possibly due to an aversion to the risk of the financial loss associated with purchasing an unwanted item.

It has been argued that laboratory environments elicit unrealistic behavioral and neural responses and, as such, findings might not be generalizable to real-world scenarios (Brofenbrenner, 1977; Ladouce et al., 2017). In particular, the limiting environmental conditions could hamper important aspects of embodied human cognition that are essential to the decision-making process, such as the interactions between perception, cognition and action that occur in complex natural environments (Gramann et al., 2014; Ladouce et al., 2017). As such, to gain a more encompassing and realistic insight into economic value based decisions for products, it is essential to examine these processes whilst subjects navigate a real world environment, moving toward analyzing brain responses during a realistic shopping experience (Pradeep, 2010; Minguillon et al., 2017). Owing to recent advancements in mobile EEG technology and signal processing techniques, it is now possible to examine neural responses while subjects move freely in the real world (Gwin et al., 2010; Jungnickel and Gramann, 2016; Banaei et al., 2017). As a case in point, Soto et al. (2018) used mobile EEG and eye tracking techniques to demonstrate the feasibility of using EMRPs to examine faces and objects within a valuation context in naturalistic conditions. The current study represents an initial attempt to examine economic value related ERPs in naturalistic settings where purchase decisions would be made, such as in a supermarket or shop.

The aim of the current experiment was to determine whether it is possible to resolve the spatio-temporal neural responses that encode the economic value of everyday household products during free viewing in a naturalistic, economically valid environment. A mobile EEG system was utilized to examine brain responses to products whilst subjects viewed and selected the objects that they would be most or least likely to purchase in the gallery setting. Eye tracking was used in tandem for real-world triggering and the procedure was based on a recent experiment by Soto et al. (2018). The BDM auction (Becker et al., 1964) was utilized to elicit WTP for products, which participants could freely inspect beforehand in a mock gallery.

## Materials and Methods

### Participants

Twenty-eight healthy subjects (14 females) were recruited for the current experiment. Nine subjects were removed from the final data analysis due to poor signal quality in EEG recordings (six subjects) or insufficient eye tracking data, such as a missing light emitting transistor–transistor logic (TTL) trigger in the world view camera (three subjects). The amount of data loss in the current study can be attributed to the difficulties associated with acquisition of mobile EEG data in naturalistic conditions. For instance, some free movements in the real world do not follow a stereotyped pattern and, as such, cannot be extracted using principal component analysis (PCA) or ICA methods (Jungnickel and Gramann, 2016). Data loss can also be attributed to difficulties associated with the collection of eye movement data. A TTL light emitting trigger box was used for synchronization of the data streams and, on three occasions, the light was not registered either due to misplacement of the light relative to the world view camera (two subjects), or due to a computer buffering error (one subject). The final sample consisted of 19 subjects (seven females) with an average age of 25 ± 5.02 (mean ± SD) years, three of which were left handed. All subjects were provided with information about the nature of the experiment and gave written informed consent prior to the commencement of the experiment. Full ethical approval was obtained from the University of Liverpool Research Ethics Committee, and all experimental procedures were conducted in accordance with the Declaration of Helsinki. Subjects received a £10 reimbursement for their time and an average of £12.26 ± 1.96 (mean ± SD) was retained from the £16.00 endowment given during the BDM task, which was added to their final payment.

### Product Images

The stimuli used in the current experiment consisted of 198 color images of everyday household items from a shopping catalog (2016, December 12). The images were split into three distinctive value categories; images of low value products costing between £0.35 and £2.80, images of medium value products costing between £3.00 and £5.50 and images of high value products costing between £6.00 and £8.00, with a total of 66 images in each value category. All stimuli were pseudo-randomly distributed within their value conditions for all tasks. Due to the pseudo-random distribution of products around the fixation cross combined with the fact that value categories were subjectively defined, i.e., one product could be considered high value for one subject and low value for another subject, the researchers did not anticipate any order effects of value category, and this was not analyzed in the current experiment. Furthermore, an effort was made to ensure that no two products of the same semantic category were displayed on the same board, i.e., it did not contain two toasters. Most subjects tended to view the products beginning at the top middle image on a panel and sequentially viewing the products in a clockwise manner as this tends to be the easiest method in order to remember which products have already been viewed.

**FIGURE 1:**
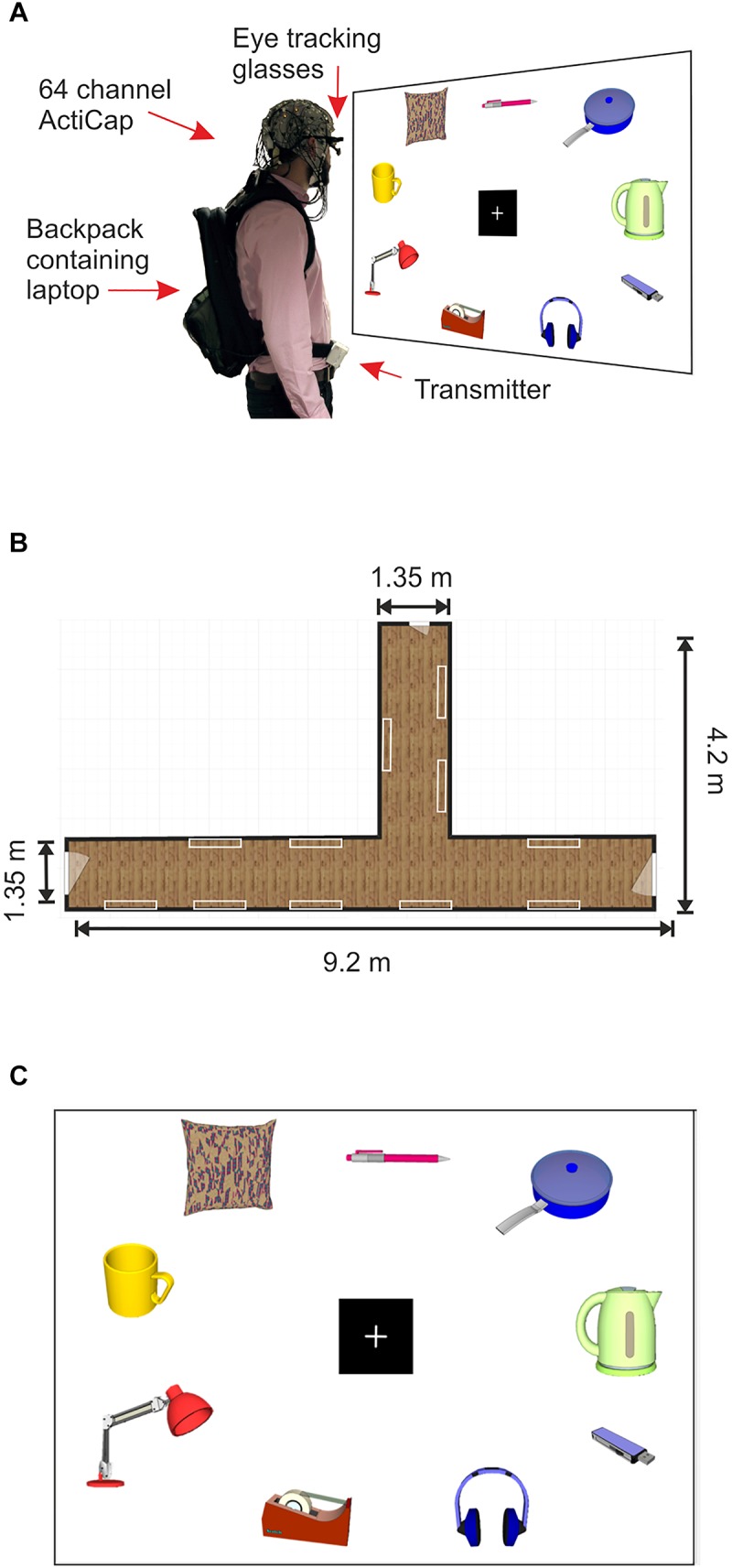
Mobile EEG set up and product gallery. **(A)** Example of a subject wearing the 64 channel actiCAP with active shielded electrodes attached to the Mobile EEG transmitter, located on the subject’s belt. The subject also wears PupiLabs eye tracking glasses which are plugged into a laptop located in a backpack. Subject looks at product panel. **(B)** Schematic representation of the corridor where the product gallery task took place. The white rectangles represent the locations of the product panels on the walls. **(C)** Schematic representation of a product panel located within the gallery. Product images in the figure were created using 3D Warehouse templates in SketchUp 2018. Each panel contained nine images of household products from a shopping catalog. The nine images were divided into three images from three different retail price categories, high (£6.00–£8.00), medium (£3.00–£5.50), and low (£0.35–£2.80).

All images were presented on 22 A0 sized poster sheets which were mounted on to Styrofoam panels of equal size using adhesive tape (Figure 1A). Each panel displayed three images from each value category (low, medium and high) with a total of nine images per panel. All panels were mounted on to the walls of two hallways within a building at the University of Liverpool using adhesive Velcro, creating a product gallery setting (Figures 1A,B). All images (sized at around 15 cm × 20 cm) were arranged around a central fixation cross (14.3 cm × 14.3 cm, see Figure 1C). Across all panels, the accumulative value for all objects on each panel ranged between £32.30 and £42.24, with a mean price of £38.16 ± £0.53 (mean ± SD).

### Procedure

The experiment was split into two separate sessions due to the time intensive nature of the mobile EEG and eye tracking set up. Session 1 included the product gallery task followed by the BDM auction task, and session 2 consisted of a behavioral rating task for hedonic aspects of the stimuli. In the first experimental session, subjects arrived at a laboratory in a building at the University of Liverpool. Instructions were delivered and full informed consent was obtained. Participants had their heads measured and were then fitted with an EEG cap (actiCAP, Brain Products, GmbH). Electrolyte gel was applied to the scalp using a syringe and 64 electrodes were then attached to the cap, with care taken to ensure that electrode impedances were kept under 50 kΩ. Following impedance checks, subjects were connected to the mobile EEG system and wireless signals were visually inspected during movement. Eye tracking glasses (Pupil Binocular Eye Tracking Glasses, PupiLabs, Germany) were then fitted to the participant over the EEG cap and plugged into a lightweight Lenovo laptop using a universal serial bus connector. The subject’s gaze was manually calibrated against a blank A0 sized panel using a 3D calibration routine with manual markers kept at a distance of 1 m.

The laptop was placed in a backpack which was worn by the participant during the mobile part of the experiment. The EEG electrode cables that ran from the EEG cap to the MOVE system transmitter (MOVE, Brain Products, GmbH, Mnich, Germany) on the subject’s belt were clipped on to the backpack in order to reduce the likelihood of generating cable sway artifacts during gait (Gramann et al., 2010; Gwin et al., 2010, see Figure 1A for an example of the set up). The MOVE system receiver, amplifier and battery were plugged into a Toshiba laptop and all of the equipment was seated on a mobile trolley, which the experimenter pushed during the experiment. If the subject was moving too far out of range of the cart, the experimenter would push the cart closer to the transmitter in order to maintain optimal signal. As the subject was moving freely, it is possible that the distance between the subject and the receiver varied over the course of the experiment, however, the raw signal was consistently monitored throughout the experiment, and the distance between the transmitter and receiver never exceeded 7 m, after which the signal to noise ratio (SNR) is known to deteriorate (Reis et al., 2014).

Likewise, impedances were lowered to 50 kΩ at the beginning of the experiment and were checked in the break between the experimental blocks, as impedances can change during movement. If individual electrodes became noisy during the experiment, they were corrected using electrolyte gel while the subject made their ratings on an A4 sheet of paper, so as to distract them as little as possible during the task. Examination of whether there was any temporal modulation of the SNR over time during the experiment was also conducted by computing the broadband spectral power in the data at three time bins in block one and block two for all subjects. No significant modulation of the broadband spectral power of the data was observed (all *p-*values > 0.05), which suggests that the signal quality was maintained throughout the experiment.

The product gallery task consisted of two experimental blocks and took approximately 30 min to complete. Following the product gallery task, the EEG equipment and eye tracking glasses were removed and participants then took part in BDM auction task for the same 198 products in a laboratory space at the University of Liverpool. The task took approximately 35 min to complete. The second part of the experiment took place within 7 days of the first experimental session. Subjects arrived in the same laboratory space at the University of Liverpool and took part in a behavioral rating task for the same 198 products. This task took approximately 35 min to complete. Afterward, subjects received two random items that they had bid on and won in the BDM auction task, were asked to fill in a payment form and were debriefed and thanked for their time.

### The Product Gallery Task

Following the mobile EEG and eye tracking set up in the first session, subjects took part in the product gallery task, which was conducted in two adjoining corridors at the University of Liverpool (see Figure 1B). At the beginning of both blocks of the product gallery task, a light emitting trigger box was used in order to synchronize EEG and eye tracking data sets. In both blocks, subjects were instructed to move in a natural manner through the gallery. Subjects were informed that they could view the panels and the images on the panels in any order, provided that they viewed all of the images on one panel before continuing on to the next. Subjects were also instructed to look at each object for a minimum of 3 s, and should always return their gaze to the fixation cross in the center of the panel before moving on to look at another image. After viewing a complete panel, subjects were required to indicate two objects that they would be most likely to buy and two objects that they would be least likely to buy on an A4 version of the panel using the pen provided. Importantly, to produce a more natural environment, the corridor was not isolated from the rest of the building and people were free to walk past at any point.

The decision to create a product gallery rather than use actual 3D products was to try and observe, for the first time, whether eye movement related potentials to EMRPs for products could be recorded outside of a laboratory-based environment at all before moving on to create a more ecologically valid setting in which 3D objects are evaluated. By using product images, it was possible to introduce some level of control into the experiment, while still maintaining a naturalistic gallery-like scenario. This allowed researchers to standardize the front facing view of the objects, the size of the objects, the lighting, and other aspects of the stimuli that could have influenced ERPs, to see whether it was possible to record ERPs at all in this naturalistic context.

### The BDM Auction Task

Following the product gallery task, subjects took part in a BDM auction task, which was displayed on a Dell monitor using a HP Compaq 8200 Elite computer. Presentation of the stimuli was controlled using Cogent 2000 (UCL, London, United Kingdom) running on MATLAB (version R2014a, The MathWorks, Inc., United States). The BDM task (Becker et al., 1964; Wilkinson and Klaes, 2012) was adapted from previous studies (Plassmann et al., 2007, 2010; Tyson-Carr et al., 2018). During the auction, 198 images of everyday household products from a shopping catalog were presented once. Each trial in the auction task was comprised of a fixation cross (presented for 2 s), followed by an evaluation stage for the product (image presented for 3 s) and then a bidding stage (presented until button press) where participants were required to bid the amount they would be willing to pay for the product. Participants were required to bid between £0 and £8 on the products in increments of £0.50 and increments of £1 from £3.00 onward, producing a total of 11 bidding options.

During the feedback stage, subjects were notified as to whether the item was ‘purchased’ or ‘not purchased.’ The purchasing outcome was dependent on the subject’s bid and its relationship with a randomly generated number. An item would be purchased if *b* ≥*r*, where *b* represents the subject’s bid and *r* represents the randomly generated number for each individual trial. Afterward, two ‘purchase’ auction trials were randomly selected, and for both items, the corresponding price of *r* was deducted from the subject’s endowment of £16, which covered a maximum of £8 being spent on each trial. The subject received these winning items during the second experimental session.

### Behavioral Rating Task

In the second experimental session, which took place within a week of the first experimental session, subjects took part in a behavioral rating task in the same laboratory space at the University of Liverpool. Presentation of stimuli was again controlled using Cogent 2000 (UCL, London, United Kingdom) running on MATLAB (version R2014a). During the task, subjects provided hedonic ratings for the same 198 product images using two sliding visual analog scales (VAS), which were sized at 10 cm and were anchored from ‘not desirable’ to ‘very desirable’ and from ‘unpleasant’ to ‘pleasant’. Each trial consisted of a fixation cross (presented for 1 s), followed by an evaluation stage (presented for 2.5 s) and, finally, a rating screen (presented until button press). This task took approximately 35 min to complete.

### EEG Recordings

EEG was continuously recorded over the whole scalp using a 64 channel wireless mobile EEG system (MOVE, Brain Products, GmbH, Münich, Germany). The wireless system included a lightweight signal transmitter which was carried by the subject on a Velcro belt tied around their waist, and a signal receiver, which was connected to the EEG amplifier and battery (see Figure 1A). Active Ag/AgCl electrodes were connected to the scalp via an elastic cap (actiCAP, Brain Products, GmbH) according to the 10-20 electrode system, using electrolyte gel to ensure electrode-to-skin impedances were consistently kept under 50 kΩ (SignaGel, Parker Laboratories, Inc., Fairfield, NJ, United States). EEG recordings were sampled at a rate of 1,000 Hz, with electrode FPz used as the system ground and all electrodes were referenced to Fz. The EEG cap was placed in accordance with the midpoint of the anatomical landmarks of the nasion, the inion and the left and right pre-auricular points. EEG average reference was applied to all electrodes and signals were digitized to 1 kHz on a BrainAmp DC amplifier running on Brain Vision Recorder version 1.20.0601 for Windows on a Toshiba Satellite P875-149 laptop. A 50 Hz notch filter was utilized during the recording.

Given that the current study was exploratory in nature, high density recordings were necessary in order to investigate not only the temporal sequencing of economic decision making, but also to spatially estimate which brain regions were activated during decision making. Furthermore, high density EEG systems afford the use of advanced computational methods such as ICA to remove many artifacts that contaminate the data, as the more channels that are provided, the more effective ICA is at separating cerebral from non-cerebral artifacts (Palmer et al., 2008; Gramann et al., 2010; Gwin et al., 2010; Lau et al., 2012). A 64 electrode system represented a compromise between high density recordings in naturalistic environments, and more quick, convenient and wearer-friendly experimental set ups.

### Eye Tracking Recordings and Analysis

Eye tracking recordings were taken on Pupil Binocular Eye Tracking Hardware using Pupil Capture software (version 0.9.6) running on Ubuntu SMP for Linux on a Lenovo Thinkpad x250 Ultrabook laptop (see Figure 1A). Both eye cameras and the world view video data streams maintained 800 × 600 resolution. The sampling rate for the world view camera was set at 60 Hz and the eye cameras were sampled at 120 Hz, however, the actual sampling rate of the world view camera was calculated offline to be 48.29 Hz (± 2.58) on average across all subjects. The pupils of both eyes were detected using a plugin for Pupil Capture software that algorithmically separates the pupil from the cornea (center-surround detection algorithm, Swirski et al., 2012). A manual 3D calibration method was employed, whereby a grid of a minimum nine points was generated on a blank A0 sized panel in the world view camera of the subject. This protocol was repeated until gaze positions were ascertained to be accurate at all points where stimuli occurred on the panel. Mid-recording calibrations were conducted if pupil gaze was lost or misaligned during the product gallery recording.

The video streams were then exported and eye tracking data was subsequently processed using the Pupil Player Program (version 0.9.6). If the gaze fixation marker was off center, fixation offsets were manually corrected using the Manual Gaze Correction plugin and fixation jitters were accounted for using in-house scripts in MATLAB version R2014a. Raw gaze positions were exported using the Raw Data Exporter plugin. Raw data exported files contained gaze positions, eye positions and level of confidence for each individual frame, as well as a corresponding time stamp based on the computer’s real time clock. Eye tracking videos were then visually inspected and the onset for a stimulus was defined as the first instance in which the gaze touched any part of the stimulus. The image reference number, value level and the onset frame was manually tabulated into an excel spreadsheet. Two subjects’ data were excluded from the sample due to loss of gaze calibration during the recordings. Analysis of gaze duration was not included as no information was registered for the last instance in which the subjects gaze left the object. This was due to researchers only being interested in using gaze onset for real-world triggering. Subjects were asked to view each product for a minimum of 3–4 s, and could continue viewing the products for as long as they liked.

### EEG and Eye Tracking Data Synchronization

A trigger box with a light emitting diode was used to temporally synchronize the eye tracking and EEG data streams. A pulse of light was delivered into the world view camera whilst a TTL pulse was inputted into the continuous EEG data and, from this, the frame in which the pulse of light was offset in the eye tracking data and the last TTL trigger registered in the EEG data was recorded and used to zero both clocks. The temporal accuracy of the synchronization trigger was tested previously in a 15-min recording whereby 15 synchronizing light/TTL pulses were produced every minute and the temporal asynchrony between triggers in both data streams was 0.022 ± 0.020 ms (mean ± SD) within a 15-min period.

Using a custom Matlab script, subject’s BDM ratings were split into quartiles based on monetary values assigned to the products, producing four SV levels: low value, low medium value, high medium value, and high value, and these were used to retrospectively redefine the value conditions. These subjective BDM values for each object were combined with the timestamp of the computer’s real time clock that corresponded to the tabulated frames where the eye first hit each object, and this was combined with a set file for each block in order to create an event file to import the triggers into the EEG data. In this way, value conditions were defined by each individual subjectively rather than by retail price.

### Eye Movement Related Potentials and Handling Eye Movement Artifacts

EEG data was pre-processed using Brain Electrical Source Analysis (BESA) software version 6.1 (MEGIS Software GmbH, Munich, Germany). The data was referenced to a common average (Lehmann, 1987), and, following visual inspection, eye blink artifacts were identified by defining their topographies and removed using a principle component analysis pattern selection algorithm which identifies artifacts based on topographies of marked segments and excludes them from the data (Berg and Scherg, 1994). Muscle artefacts were manually selected and removed from the data. Event markers were inserted into the data by temporally synchronizing the EEG and eye tracking data sets using custom Matlab scripts. The time period for baseline correction was from -300 ms to 0 ms, and the data was epoched from -300 pre-stimulus to 600 ms after the instance when the eye first hit the object (0 ms). The data was filtered from 1 to 35 Hz and all time locked post-saccadic EMRPs from all subjects across four value conditions (low, low medium, high medium, and high value) were analyzed.

Due to the time locking of EMRPs to the offset of saccades, a number of saccade related artifacts needed to be extracted from the data. Saccade related artifacts are generated by rotation of the corneoretinal dipole of the eye (Berg and Scherg, 1991; Dimigen et al., 2011), movement of the eyelid during blinking and vertical or horizontal saccades (Picton et al., 2000; Dimigen et al., 2011) and muscular activation at the beginning of a saccade, referred to as the saccadic spike potential (Thickbroom and Mastaglia, 1986; Dimigen et al., 2011; Nikolaev et al., 2016).

To separate further eye movement artifacts such as saccade-related potentials from genuine cortical activity, an infomax ICA analysis (Jung et al., 2000; Iriarte et al., 2003; Khushaba et al., 2013; Nikolaev et al., 2016) was performed which algorithmically separates the grand average signal into its maximally statistically independent constituents. An infomax ICA was conducted using concatenated grand averaged data from four different value conditions (2,400 time points). ICs weights were estimated, and, of these, ICs were selected based on spatial and temporal properties as well as responsiveness to value conditions. Subsequently, individual ICs were back projected onto single subject average data by loading the grand average ICA weights on single subject averages and exporting only the individual IC data of interest (Debener et al., 2010). This method allowed for the removal of ICs that represented residual saccadic artifacts from the grand averaged sensor signal by only back projecting the ICs of interest.

### Source Dipole Modeling

To localize the generators of cortical potentials represented in ICs of interest, IC waveforms were analyzed using source dipole analysis in BESA version 6.1 program. Using a sequential strategy (Stancak et al., 2002; Hoechstetter et al., 2010), equivalent current dipoles (ECDs) were fitted to describe the 3-dimensional source currents in the regions contributing predominantly to the data (Scherg and Von Cramon, 1986). ECDs were fitted one at a time to explain the latency components starting with the shortest latency. ECDs had free origins and orientations. The fitting procedure was stopped when the ECD explained the maximum amount of variance (at least 90%) or if the dipole was located outside of the head. A 4 shell ellipsoidal volume conductor model was used to create the source dipole model with the following conductivity levels assumed; head = 0.33 S/m, scalp = 0.33 S/m, bone = 0.00 S/m, and cerebral spinal fluid = 1.00 S/m.

### Statistical Analyses

For behavioral ratings, separate one way repeated measures ANOVAs (four levels) were employed to examine the relationship between value level (as defined by BDM rating) and BDM rating, retail price, desirability and pleasantness ratings. Greenhouse-Geisser corrections were used to overcome the violation of sphericity assumption when necessary. All significant effects were further analyzed using *t*-tests and a critical threshold of *p* < 0.005 was utilized. All standard statistical tests were carried out in SPSS v. 24 (IBM Corp, 2016).

Independent component analysis waveforms for each individual IC were exported and one way repeated measures ANOVAs were conducted using the EEGLab toolbox (Delorme and Makeig, 2004). The four SV levels (low value, low medium value, high medium value, and high value) were compared against IC amplitude across time windows where amplitude was maximal for each IC. *T*-tests were also used to compare all low versus all high value conditions for each IC of interest. A 95% confidence level was always employed. To reduce the likelihood of generating false positives, *p*-values were corrected using 1,000 permutations (Maris and Oostenveld, 2007) and a critical threshold of *p* < 0.005 was utilized.

## Results

### Behavioral Results

**FIGURE 2:**
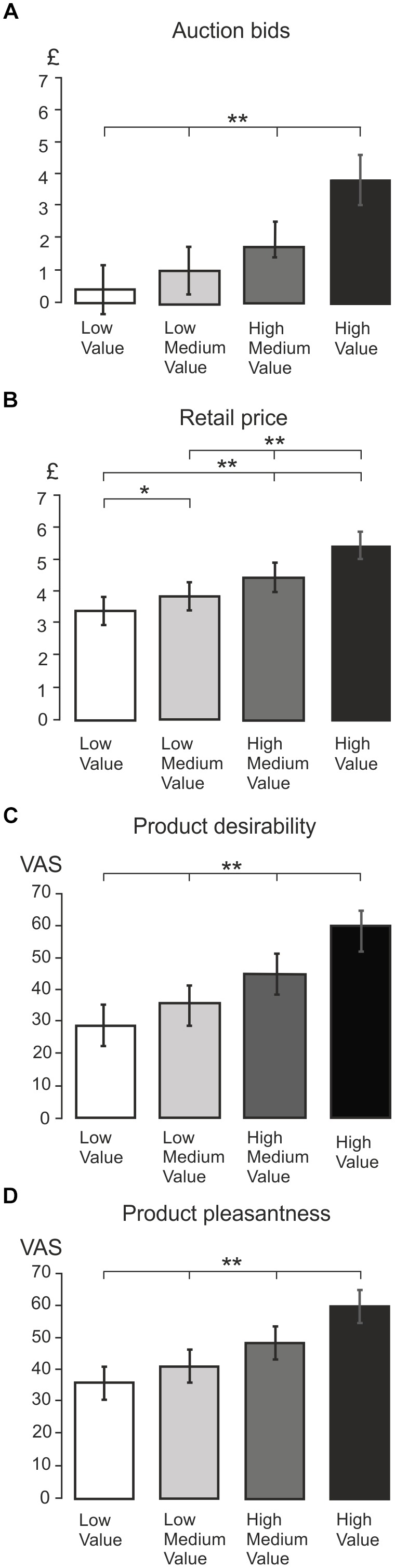
Average behavioral ratings. **(A)** Bar graph showing the mean BDM auction bids for four different value levels: low, low medium, high medium, and high value. The value conditions are split by subject’s auction rating, and the bar graph shows significant differences between all value conditions. A double asterisk (^∗∗^) indicates presence of high statistical significance (*p* < 0.001), and a single asterisk indicates statistical significance (*p* < 0.05). This provides validation for the splitting product stimuli into four value categories based on BDM auction value. **(B)** Bar graph showing mean retail price across the four BDM auction value conditions. Highly significant differences were indicated with a double asterisk, and *p* < 0.05 was indicated with a single asterisk. The bar graph indicates that significant differences were found in retail price across all value levels (*p* < 0.05), suggesting that BDM auction value ratings mirror the actual retail price of the product. **(C)** Bar graph showing mean product desirability rating across four value conditions. From the graph it can be seen that mean desirability increased incrementally with BDM value (all *p* < 0.001). **(D)** Bar graph showing mean product pleasantness rating across all value levels. Again, this graph shows that mean pleasantness increases incrementally with BDM value (all *p* < 0.001).

Figures 2A–D show the mean values of WTP, retail price, desirability and pleasantness in four different levels of values ranging from low to high value, respectively. All of these measures showed a statistically significant relationship with SV level according to one-way ANOVAs for repeated measures with four levels of values as the independent variable [BDM: *F*(1,24) = 141.22, *p* < 0.001; retail price: *F*(2,43) = 72.61, *p* < 0.001; desirability: *F*(1,37) = 89.13, *p* < 0.001; pleasantness: *F*(2,38) = 75.53, *p* < 0.001]. In all dependent measures, the *t*-tests showed statistically significant differences across all value levels (*p* < 0.005). Additionally, there was a highly significant linear trend component (*p* < 0.001 in all cases), confirming a linear increase in WTP, retail price and subjective ratings across all SV categories.

### Eye Movement Related Potentials

**FIGURE 3:**
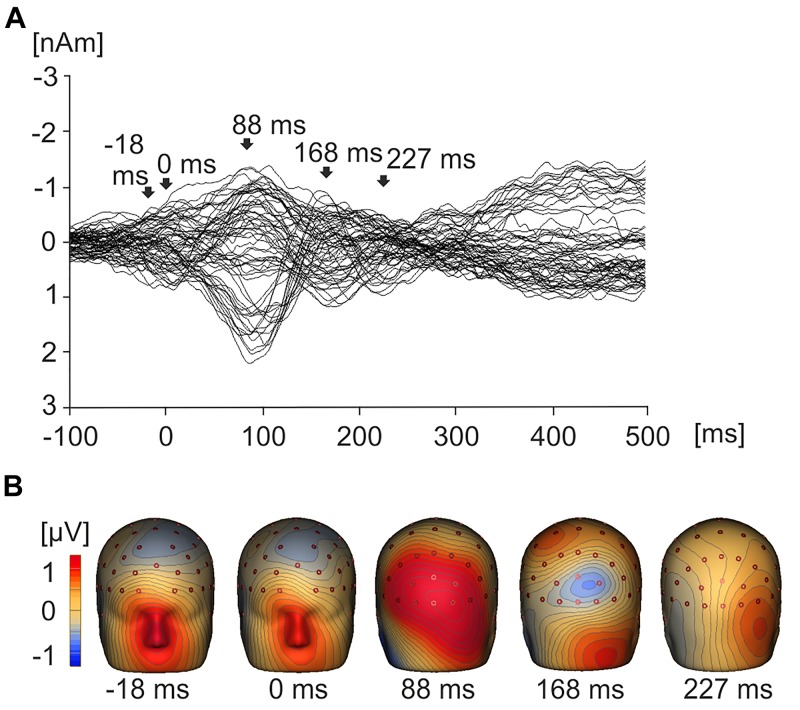
Grand average EMRPs waveforms and topographic maps. **(A)** Butterfly plot showing grand average EMRPs waveforms across all subjects and all product value conditions with key points of interest highlighted with arrows. The butterfly plot demonstrates that eye movement activity is present in the baseline (–18 ms) and residual eye movement is present when the eye first touches the image (0 ms). The lambda component is highlighted (88 ms) and two later value related peaks are observed at 168 ms and 227 ms. **(B)** 3D whole head topographic maps displaying grand average EMRP cortical activation at key time points (–18 ms, 0 ms, 88 ms) and value related peaks (168 ms and 227 ms).

Figure 3A displays a grand average butterfly plot demonstrating EMRPs across all value conditions and all 19 subjects. Figure 3B illustrates the topographic maps corresponding to time points of interest that are highlighted in Figure 3A. During the pre-stimulus interval prior to onset of fixation, the topographic map displays a large frontal positivity which is maximal in the region of the eyes at -18 ms across all conditions and subjects. This potential component represented a corneoretinal artifact, and was associated with the offset of the saccadic eye movement when the subject directed their gaze toward a particular stimulus. At stimulus onset (0 ms), there was residual corneoretinal artifact associated with a saccadic eye movement. The lambda potential (Yagi, 1979, 1981; Thickbroom et al., 1991) (Figures 3A,B) peaked at 88 ms and demonstrated a large positivity across occipital electrodes similar to P100 component in a visual evoked potential. Figure 3A also demonstrates a positive peak around 168 ms, which was associated with positivity in parietal electrodes (Figure 3B). Another peak emerging at 227 ms demonstrated bilateral posterior positivity (Figures 3A,B).

### ICA Reconstruction of Eye Movement Related Potentials

**FIGURE 4:**
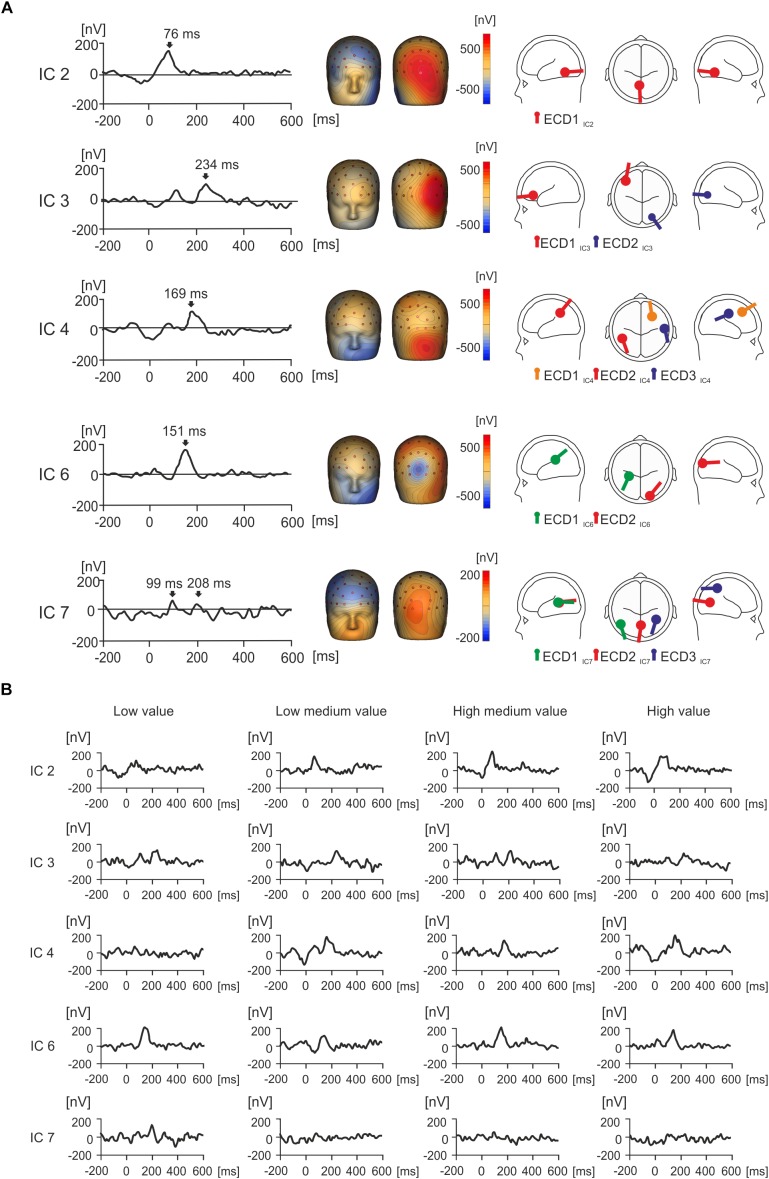
Grand average IC activity (nV) and value. **(A)** Grand average waveforms for each isolated IC of interest between –200 and 600 ms, with peak activity for each component indicated with an arrow. Source dipole modeling was used to estimate equivalent current dipoles (ECDs) in order to explain the cortical sources of activation for each IC. ECDs for each IC can be observed in a glass brain showing the location and orientation of estimated cortical activity, with no more than three sources utilized. **(B)** The grand average waveforms for each individual IC were split by condition in order to illustrate how each IC responds to value over time.

Figure 4A shows the grand average IC activities for five separately back-projected IC components collapsed across four value conditions (low, low medium, high medium, and high value products). Figure 4A also shows the topographic maps and source dipole solutions for each of the ICs. Figure 4B demonstrates how individual IC amplitude responds separately for each of the four value conditions.

IC2 showed a strong positive peak at 76 ms, and the topographic maps presented a strong positive potential in the right occipital region of the scalp (Figure 4A). This spatio-temporal pattern was modeled with one ECD (ECD1_IC2_) which was fitted in the visual association area (Brodmann area 18, approximate Talairach coordinates *x* = -4.5, *y* = -56.3, *z* = -10.4 mm). The peak in IC2 was seen in all four value conditions (Figure 4B).

IC3 displayed a positive potential maximum in the right occipito-temporal electrodes and a negative potential in the left frontal region of the scalp (Figure 4A). The time course of IC3 manifested a peak at 112 ms followed by a double peak around 210 ms and later around 250 ms. The spatial pattern of IC3 was modeled with two ECDs. ECD1_IC3_ explained the most variance for left frontal negative activation in the dorsolateral prefrontal cortex (Brodmann area 46, approximate Talairach coordinates *x* = -41.7, *y* = 37.1, *z* = 5.4 mm). ECD2_IC3_ accounted for the right occipital positivity and was placed in the visual association area (Brodmann area 19, approximate Talairach coordinates *x* = 24.9, *y* = -78.9, *z* = -4.4 mm). While the later latency peak at 250 ms was seen in all four value conditions, the earlier peak (212 ms) was prominent only in the low- and high-medium value conditions (Figure 4B).

IC4 showed a peak at 169 ms (Figure 4A). The spatio-temporal maps of IC4 showed two large positive and negative component maxima in frontal and occipital regions of the scalp, respectively, and further positive maxima in centroparietal electrodes. This complex spatio-temporal configuration required a model with three ECDs (Figure 4A). ECD1_IC4_ explained the large negative potential maxima in the left frontal region and was located in the frontal eye field area of the cortex (Brodmann area 8, approximate Talairach coordinates *x* = 28.8, *y* = 20.6, *z* = 44.7 mm), which was maximal around 169 ms. ECD2_IC4_ was fitted into the left parietal area (Brodmann area 39, approximate Talairach coordinates *x* = -45.9, *y* = -50, *z* = 33.4 mm), to explain the temporal positivity. ECD3_IC4_ was fitted to the right primary somatosensory cortex (approximate Talairach coordinates *x* = 56.3, *y* = -16.7, *z* = 35.8 mm) and explained right localized parietal negativity and right occipital positivity at 169 ms. The IC4 component peak at about 169 ms was seen in all value conditions except the low-value condition (Figure 4B).

IC6 demonstrated a positive peak occurring at 151 ms (Figure 4A). The topographic map manifested a frontal negative potential, a parietal positivity, and a localized negative potential in the midline occipital electrodes. Two ECDs explained this topographic map. ECD1_IC6,_ located in the parietal cortex (Brodmann area 40, approximate Talairach coordinates *x* = -28.4, *y* = -32.7, *z* = 21.5 mm) accounted for frontal negativity. ECD2_IC6_ pointed to the negative potential in the midline occipital electrodes (Brodmann area 18, approximate Talairach coordinates *x* = 22.5, *y* = -97.0, *z* = 9.4 mm) and was located in the right visual association area. The peak at about 150 ms was seen in all four value conditions (Figure 4B).

Figure 4A displays a peak for IC7 at 99 ms, and a second smaller peak at 208 ms, with large frontal negativity and occipital positivity. Three ECDs were used to explain this activation, and this can be seen in Figure 4A. ECD1_IC7_ explained most variance in the frontal cortex (Brodmann area 19, approximate Talairach coordinates *x* = -48.7, *y* = -65.9, *z* = 6.7 mm), which peaked at 208 ms and accounted for negativity in the frontal cortex. ECD2_IC7_ explained most variance in the left occipital region (Brodmann area 18, approximate Talairach coordinates *x* = -0.3, *y* = -71.2, *z* = 7.3 mm), in the left primary visual area, peaking at 208 ms and explaining the occipital positivity. ECD3_IC7_ explained a source in the parietal cortex (right Brodmann area 39, approximate Talairach coordinates *x* = 39.4, *y* = -55.8, *z* = 44.9 mm), in the angular gyrus, and this accounted for right frontal negativity peaking at 208 ms. In Figure 4B, IC7 displays a peak for low value objects at 208 ms that does not seem to appear for other value conditions.

**FIGURE 5:**
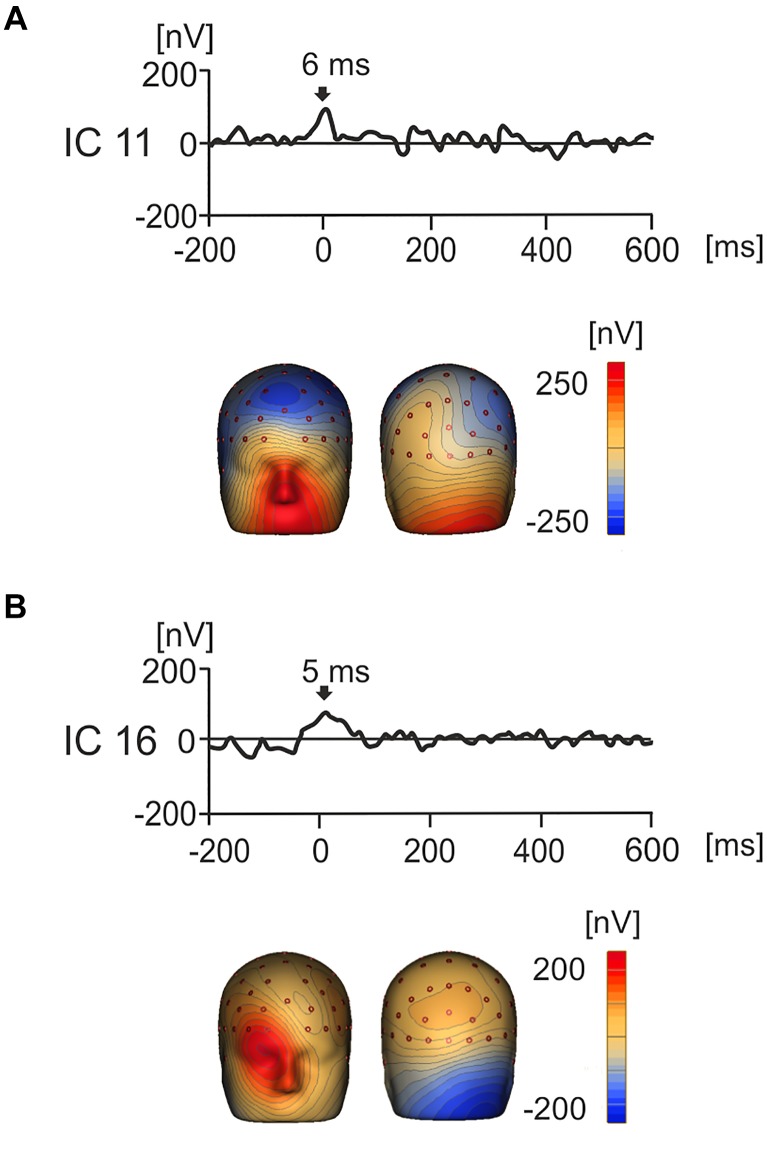
Grand average IC waveforms and corresponding 3D whole head topographic maps (nV) for two ICs that represent eye movement related artifacts. **(A)** IC11 showed peak activity around image onset (6 ms), with a positive maxima around the eyes, suggesting that this component represented left biased saccadic eye movement related activity. **(B)** IC16 showed peak activation around product image onset (5 ms) and positivity maximal around the right eye, suggesting that that this component represents right-biased saccadic eye movement related activity.

Notably, the use of ICA afforded the separation of components that had a cerebral origin and responded to product values from the potentials caused by oculomotor activity such as saccades, spike potentials and residual eye blinks (Thickbroom and Mastaglia, 1986; Berg and Scherg, 1991; Picton et al., 2000; Dimigen et al., 2011; Nikolaev et al., 2016). Examples of the artifact-related ICs are shown in Figures 5A,B. For instance, IC11 showed strong positive activation around the eyes which peaked at 6 ms indicating that this IC represents artifactual saccadic activity. Likewise, IC16 showed a positive potential maximum that was biased to the right eye with a peak at 5 ms. This suggests that the subject was making a right sided saccade when their gaze touched the first image.

### The Effect of Value on ICs

**FIGURE 6:**
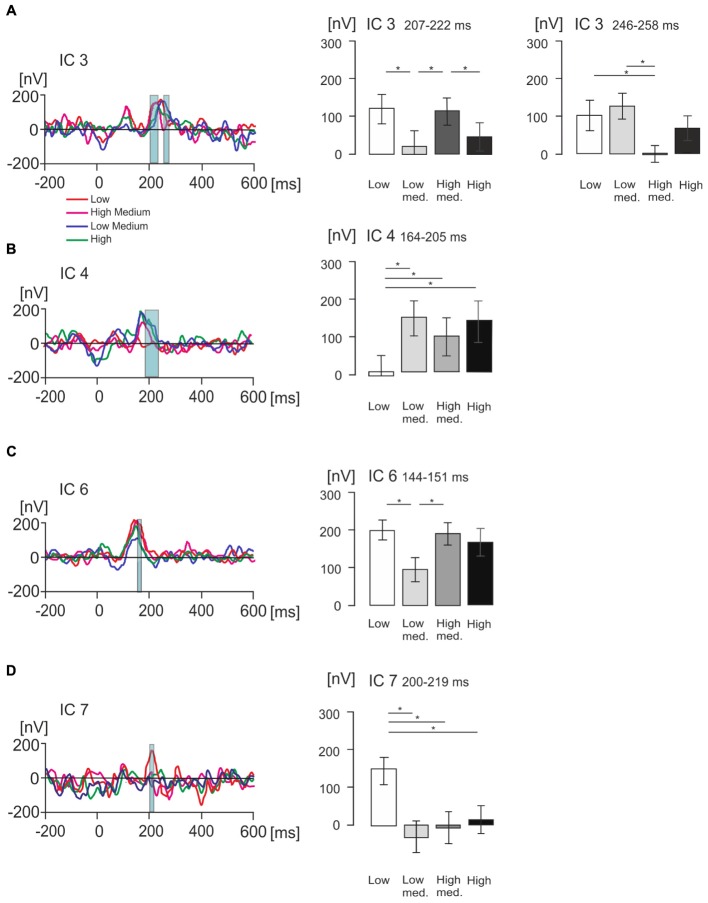
Statistical differences in product value for ICs of interest measured in nanovolts (nV). **(A–D)** Figures illustrating grand average EMRP activity split across four value conditions, indicated by different colored lines, for four isolated ICs of interest including IC3, IC4, IC6 and IC7 respectively. The corresponding bar graphs illustrate differences in mean amplitude across the four subjective value conditions for each isolated IC of interest. Significant differences between mean amplitude across conditions are indicated with a single asterisk for differences significant at *p* < 0.05.

Figures 6A–D show, for each individual IC, statistically significant effects of values with all value conditions superimposed and bar graphs with mean voltage amplitude differences at key time points of interest for each IC, with standard error bars. IC3, IC4, IC6, and IC7 showed statistically significant effects of value categories according to a one-way ANOVA for repeated measures which was conducted for five components, including IC2, across all time points ranging from -200 ms to 600 ms.

In IC3, a statistically significant effect of value was found in two latency intervals, 207–222 ms and 246–258 ms. In the time window 207–222 ms [*F*(2,42) = 7.22, *p* < 0.005, Figure 6A], the effect of product value was largely driven by the low medium value products demonstrating a significantly lower amplitude compared to low (*p* = 0.014) and high medium products (*p* = 0.006). The high-medium value also demonstrated a significantly higher IC3 amplitude than the high value category (*p* = 0.033). When all low and all high value conditions were compiled, there were no statistically significant differences (*p* > 0.05).

In the latency interval 246–258 ms, the effect of product values [*F*(2,51) = 6.67, *p* = 0.001] was related to the high-medium products producing lower IC3 amplitude compared to low value (*p* = 0.013) and low medium product amplitude (*p* = 0.007). When all low and all high value conditions were compiled and compared using a *t*-test, we found low value products produced higher IC3 amplitude (116 ± 26 nV, mean ± SEM) compared to high value products (33 ± 21 nV, mean ± SEM), and this difference was statistically significant [*t*(36) = 4.02, *p* < 0.001].

In IC4 (Figure 6B), a statistically significant effect of product values was found in the latency interval 164–205 ms [*F*(2,42) = 7.36, *p* < 0.005], with low value products producing significantly smaller amplitude compared to low-medium value (*p* = 0.002), high-medium value (*p* = 0.03), and high value products (*p* = 0.04). When low and high value levels were compiled, products showed no statistically significant differences (*p* > 0.05).

In IC6 (Figure 6C), the product values differed significantly in the latency interval 144–151 ms [*F*(2,41) = 5.61, *p* = 0.005] with low value products producing significantly higher IC6 amplitude compared to low-medium value products (*p* = 0.005), and high-medium value producing significantly higher IC6 amplitude compared to low-medium products (*p* = 0.023). When low and high values were compiled, the difference between low and high value was not statistically significant (*p* > 0.05).

Finally, IC7 showed no statistically significant effect on the peak at 99 ms, but did show a statistically significant effect of product values in the latency interval 200–219 ms [*F*(2,45) = 6.95, *p* = 0.001, Figure 6D]. This effect was driven by the low value category producing significantly higher IC7 amplitude compared to low-medium (*p* = 0.009), high-medium (*p* = 0.017) and high value conditions (*p* = 0.010). When all low and all high values were compiled, the difference was not statistically significant (*p* > 0.05).

## Discussion

The present study explored the cortical representations of economic decisions for products using EMRPs extracted from mobile EEG. ICA revealed a typical lambda component as well as four components that were modulated by subjective economic value in the latency range of 150–200 ms. The most important finding in the current study was that two ICs demonstrated contrasting responses to low value products, with IC4 modulating amplitude for all except the lowest value products, and IC7 exhibiting enhanced amplitude for lowest value products.

Viewing high and intermediate value items was associated with comparatively strong IC4 activity peaking at 169 ms. This enhanced responsiveness of IC4 to all value conditions excluding low suggests that there is early enhanced attentional processing for higher valued items and this could point toward a relatively automatic valuation system that preferentially attends to more highly valued items (Anderson et al., 2011; Glimcher and Fehr, 2014). Support for this interpretation comes from research demonstrating that a shorter latency and larger amplitude of the P200 component was associated with early preferential attention (Hanatani et al., 2005). In a recent study, Tyson-Carr et al. (2018) found that the P200 component was modulated by valuation context during product preference decisions. The authors observed enhanced P200 activation when subjects considered product desirability, which they suggested was related to attention allocation during valuation decisions. These results align with the present experiment in that more highly valued, desirable stimuli received enhanced attention, making it more likely that they would be readily purchased at a later stage. Likewise, the P200 has also been associated with preferential processing of primary reinforcers such as sugary foods (Schienle et al., 2017). Our data suggests that the cortical response captured by IC4 represents an automatic attention allocation mechanism that responds preferentially to higher valued stimuli in order to facilitate purchasing for higher valued items and to avoid aversive low value stimuli, irrespective of product category. An ECD for IC4 was estimated in the left parietal area (BA39). The parietal cortex has been shown to become activated during explicit comparisons (Menon et al., 2000; Cappelletti et al., 2010; Hsu and Goh, 2016) and correlated with evidence seeking during reward related decisions (Furl and Averbeck, 2011). An ECD for IC4 was also observed in the right primary somatosensory cortex, which has shown modulation following rewards in a somatosensory task (Pessoa and Engelmann, 2010). These results lend some support to the interpretation that IC4 represents an early neural attention allocation mechanism that selectively responds to more highly valued stimuli and is insensitive to the stimuli that are the least positively valued, even in the case of everyday household products.

In contrast to IC4, IC7 responded to low value items around 200 ms. This early enhanced processing for the low value objects can be understood in terms of very low value products representing an aversive stimulus, due to the potential for the loss of monetary resources. In support of this interpretation, the P200 ERP component has been found to be modulated by emotional valence of a stimulus (Carretié et al., 2001a; Ashley et al., 2004; Huang and Luo, 2006), and to moderate attention allocation (Carretié et al., 2001b) for products of different valence (Carretié et al., 2001a; Polezzi et al., 2008). Moreover, some studies suggest that the P200 ERP component is reflective of a subjectively negative assessment (Polezzi et al., 2008). For instance, Carretié et al. (2001b) found a higher P200 amplitude and shorter latency when viewing negative stimuli, which they attributed to enhanced attention for aversive stimuli. Source dipole modeling for IC7 revealed an ECD in the visual association area, which has been shown to be modulated by the motivational relevance of a stimulus (Krawczyk et al., 2007). Another ECD was located in the angular gyrus, which is associated with numerical problem solving, (Seghier, 2013), attention allocation for salient stimuli (Gottlieb, 2007), response inhibition (Gottlieb, 2007; Nee et al., 2007; Seghier, 2013) and stimulus value (Lin et al., 2012). Angular gyrus activation has also been observed in gambling tasks, with more activation for potential losses (Minati et al., 2012). Therefore, it is likely that the angular gyrus is involved in enhancing attention toward low value stimuli to inhibit buying and avoid monetary loss. Taken together, we suggest that IC4 and IC7 reflect attention-related and aversive responses which are likely part of an early valuation system that serves to enhance attention for intermediate and high value stimuli to facilitate purchasing and to enhance attention for aversive low value stimuli in order to facilitate avoidance (Della Libera and Chelazzi, 2009). In this way, basic economic decision making appears to occur quickly in order to isolate the lowest value products and to ensure monetary resources are being economically optimized. The authors recognize, however, that IC7 represents a very small component that peaks close to baseline, therefore, any conclusions should be viewed with caution.

Two other ICs, IC3, and IC6, preferentially responded to medium value products, although these responses are more difficult to interpret. IC6 peaked at 151 ms and exhibited higher amplitude for low and high medium value products. The P150 ERP component has been associated with a basic rapid visual categorization process and it might be that it forms part of a ‘tagging’ mechanism that marks items out for later enhanced processing downstream (Kirchner and Thorpe, 2006; Nakatani et al., 2013; De Cesarei et al., 2015). In support of this interpretation, an ECD was located in the left supramarginal gyrus, which has been implicated in semantic categorization of visual stimuli (Pexman et al., 2007), and economic decisions for products (Deppe et al., 2005). Another dipole was observed in the visual association area, which has been shown to be modulated by the motivational relevance of a stimulus (Krawczyk et al., 2007).

IC3 showed statistically significant increases in activity for medium value categories in two latency windows within the range of the P200 ERP component. Further data and replications are needed to fully understand the role of IC3 in product evaluations in natural settings. However, the finding of a source contributing to IC3 in the dorsolateral prefrontal cortex suggests that this component was related to value-based decision making as important components of decision making such as WTP, moderation of risk, and top down attention have been shown to be mediated by this brain region (Plassmann et al., 2007; Hubert and Kenning, 2008; Bartra et al., 2013; Morris et al., 2014; Mahesan et al., 2016).

Our data suggests that attention or aversion reflecting SV is attributed to products during free viewing in quasi-naturalistic settings as early as 200 ms. Further support for this comes from studies of single neurons in monkey’s orbitofrontal cortex, which have been shown to respond to values and risks as early as 180 ms (Schultz et al., 1993; Critchley and Rolls, 1996; Padoa-Schioppa and Assad, 2006). The data also suggest that this early automatic valuation is mediated by a set of cortical activation patterns, none of which encodes values in a linear fashion. Rather, our results prompt the hypothesis that certain cortical regions or sub regions of larger brain areas are tuned to respond predominantly to low -or high- value items. Value-tuned brain modules that are responsible for spotting low or high value items in our environment would allow rapid categorization and prompt behavioral avoidance or approach responses.

The fast bottom-up responses for low and higher value products and the potentially more deliberative top-down responses for more ‘difficult’ medium value decisions can be better understood from the perspective of the Fuzzy Trace Theory (Brainerd and Reyna, 1990). The theory postulates that decisions in the real world are calculated based on two processes that are computed in parallel; verbatim representations, which involve automatically matching characteristics to representations stored in memory, and more meaningful top-down gist representations (Corbin et al., 2015). In the current study, it is possible that high and low value items elicit an automatic verbatim representation as they have been experienced before, whereas medium value products require more top-down deliberation and elicit a semantic ‘gist’ representation. As such, the Fuzzy Trace Theory provides explanation for the similar latencies of the ICs as they are processed in parallel and clarifies the recruitment of top-down brain areas for medium value decisions. This draws similarities to the neuroeconomic concept that multiple brain systems are involved in the computation of value (Dickinson and Balleine, 2002; Rangel et al., 2008). Daw et al. (2005) propose that different brain systems might be involved when choosing between options with different values under varying levels of uncertainty. These systems include habitual processing, which represents fast responses that are learned through trial and error, and goal-directed responses, which involve the assignment of value through outcome assessment and reward calculation for multiple options. In the current study it could be the case that the habitual processing system was employed for valuation of products that subjects had more experience of choosing between, i.e., high and low value products. Conversely, when the product is not categorized as high or low value, the goal directed system dominates as further assessments are needed in order to determine the products’ worth. The results of the current study are also highly relevant to the field of neuromarketing as it was shown that, within the first 200 ms of viewing a product, the brain already computes and assigns a value (Jones et al., 2012; Pozharliev et al., 2015; Tyson-Carr et al., 2018). As such, it appears that first impressions are very important when deciding whether to purchase a product and previous experience and expertise can influence purchase decisions.

There are, however, several limitations associated with the current study. One limitation of this experiment is that we were unable to detect EMRPs occurring later than 300 ms post stimulus. Previous research has highlighted the role of the P300 (Yeung and Sanfey, 2004; San Martín, 2012) in outcome evaluation during economic decisions. In traditional laboratory-based experiments, subjects are presented with a fixation cross followed by a stimulus in the same predefined position in order to avoid saccade related artifacts (Dimigen et al., 2011). In contrast, free viewing in the real world is a multi-and-*trans*-saccadic process in which the visual stream is constantly being updated and integrating new sensory information into continuous perceptual and cognitive processes (Dimigen et al., 2011). As a result, the new sensory information does not show a fixed phase relative to the time locked event and distortion of the signal occurs later in the EMRP (i.e., after 300 ms; Dimigen et al., 2011). This is an ongoing issue for experiments that combine free eye movements with EEG, as it is difficult to ensure that continuous visual updating does not distort the EMRP without compromising the ecological validity of the experiment. As a result, the current findings should be viewed as preliminary, and more research is needed to determine how the brain computes valuation decisions later in the decision making process. Another limitation of the current study is that source dipole modeling was used to estimate sources for EMRP activity. It must be emphasized that any conclusions drawn from source dipole modeling in mobile EEG should always be viewed with caution and any interpretations are tentative. As such, no statistics were performed on the dipole analysis, which was conducted purely for exploratory purposes. This is because of the difficulties associated with source localization in EEG data (Luck, 2005), which are exacerbated in mobile EEG data (Grech et al., 2008).

Moreover, to avoid explicitly asking subjects to provide an economic value for a product, subjects were asked to rate whether they would purchase products during the gallery task and were later required to bid on the items, and these bids were used to retrospectively define the value conditions. However, it cannot be ruled out that subjects considered other factors such as desirability and pleasantness to inform their decision in the gallery. These factors could theoretically influence attention or aversion, as our results show that desirability and pleasantness ratings echoed the economic value of the stimulus. Future studies should endeavor to isolate value related from non-value related attentional processes, although this is very difficult to achieve. Likewise, attention, aversion and early economic valuation decisions should be further explored using fMRI and fMRI informed source analysis techniques in EEG. Finally, it is possible that the limited price range could have influenced economic value responses as the current experiment failed to show a component that responded exclusively to the highest value condition. This could be because objects within the £8 price bracket are only considered to be high value within the context of products on offer. Future experiments may benefit from expanding the price range to better examine how the temporal sequencing of economic value attribution occurs in the brain.

Finally, findings from the current study can be compared with results obtained from a standard laboratory recording. The present study follows a laboratory based experiment from our research group by Tyson-Carr et al. (2018), who found, using the same BDM auction paradigm, that the N200 resolved the valuation of everyday household products, with a bias toward low value objects. The authors suggested that the modulation of the N200 for low value objects hints toward an automatic valuation system, which is similar to what was observed in the current study. Therefore, as the current study provides further support for automatic valuation of products even in naturalistic environments, this adds validity to the data. This is an extremely important and novel contribution as the current experiment was able to demonstrate the feasibility of examining EMRPs for products in naturalistic environments.

## Conclusion

In conclusion, the current experiment demonstrated that, to some extent, the neural spatio-temporal dynamics that underpin economic decisions for household products can be resolved in a naturalistic setting. Findings suggest that the EMRP parietal P200 component reflects an attention allocation mechanism that responds extremely quickly to isolate the lowest (IC7) value stimuli from all other value stimuli (IC4), as these represent important decisions in terms of maximizing economic resources. Other components responded to medium value products and may indicate a fine-grating of more difficult decisions (IC3, IC6). Overall, while none of the ICs displayed linear amplitude changes that parallel the SVs of products, results suggest that a combination of multiple ICs may sub-serve a fine-grained resolution of the subjective economic values of products. In order to fully disentangle the spatio-temporal neural processes that underpin economic decisions for products in the real world and to better understand how medium and high value products are represented, more research is needed with a broader range of stimuli.

## Data Availability

All of the raw data supporting the conclusions of the manuscript, including EEG, eye tracking, and behavioral data, will be made available to qualified researchers upon request.

## Author Contributions

AS and TG contributed to the development of the original concept behind this work. HR, AS, NF, and TG contributed to the development of the experimental design and planning of the current project. AS, NF, and TG were awarded the funding for the project. AS and VS contributed to the development of the mobile EEG and eye tracking paradigm. HR, VS, and JT-C contributed to the development of the stimuli and materials. HR and JT-C contributed to the development of the BDM auction task. HR and AS contributed to the development of the behavioral rating task and carried out the data acquisition, pre-processing, and analysis of the collected data, and data collection was aided by VS, KK, JT-C, and SC. AS developed all of the original Matlab scripts that were used for data analysis, figure creation and the synchronization of the EEG and eye tracking data sets. HR and AS produced the final written manuscript.

## Conflict of Interest Statement

The authors declare that the research was conducted in the absence of any commercial or financial relationships that could be construed as a potential conflict of interest.

## Abbreviations

BDM: Becker-Degroot-Marschak
EMRP: eye movement related potentials
IC: independent component
ICA: independent component analysis
SV: subjective value
WTP: willingness to pay

## References

[B1] Anderson B. A. Laurent P. A. Yantis S. (2011). Value-driven attentional capture. *Proc. Natl. Acad. Sci. U.S.A.* 108 10367–10371. 10.1073/pnas.1104047108 21646524 PMC3121816

[B2] Ashley V. Vuilleumier C. A. P. Swick D. (2004). Time course and specificity of event- related potentials to emotional expressions. *NeuroReport* 15 211–216. 10.1097/00001756-200401190-00041 15106860

[B3] Banaei M. Hatami J. Yazdanfar A. Gramann K. (2017). Walking through architectural spaces: the impact of interior forms on human brain dynamics. *Front. Hum. Neurosci.* 11:477. 10.3389/fnhum.2017.00477 29033807 PMC5627023

[B4] Bartra O. McGuire J. T. Kable J. W. (2013). The valuation system: a coordinate-based meta-analysis of BOLD fMRI experiments examining neural correlates of subjective value. *NeuroImage* 76 412–427. 10.1016/j.neuroimage.2013.02.063 23507394 PMC3756836

[B5] Becker G. M. Degroot M. H. Marschak J. (1964). Measuring utility by a single- response sequential method. *Behav. Sci.* 9 226–232. 10.1002/bs.3830090304 5888778

[B6] Berg P. Scherg M. (1991). Dipole modelling of eye activity and its application to the removal of eye artifacts from the EEG and MEG. *Clin. Phys. Physiol. Meas.* 12 49–54. 10.1088/0143-0815/12/A/010 1778052

[B7] Berg P. Scherg M. (1994). A multiple source approach to the correction of eye artifacts. *Electroencephalogr. Clin. Neurophysiol.* 90 229–241. 10.1016/0013-4694(94)90094-9 7511504

[B8] Berry J. Fischer G. Guiteras R. (2012). *Eliciting and Utilizing Willingness to Pay: Evidence from Field Trials in Northern Ghana. IGC Working Paper 12/0188.* Available at: http://personal.lse.ac.uk/fischerg/Assets/BFG-BDM-April-2012.pdf%5Cnhttp://www.economics.cornell.edu/jwb295/BFG-BDM-April-2012.pdf

[B9] Brainerd C. J. Reyna V. F. (1990). Gist is the grist: fuzzy trace theory and the new intuitionism. *Dev. Rev.* 10 3–47. 10.1016/0273-2297(90)90003-M

[B10] Brofenbrenner U. (1977). Toward an experimental ecology of human development. *Am. Psychol.* 32 513–531. 10.1037/0003-066X.32.7.513

[B11] Cappelletti M. Lee H. L. Freeman E. D. Price C. J. (2010). The role of the right and left parietal lobes in the conceptual processing of numbers. *J. Cogn. Neurosci.* 22 331–346. 10.1162/jocn.2009.21246 19400672 PMC2808313

[B12] Carretié L. Martin-Loeches M. Hinojosa J. A. Mercado F. (2001a). Emotion and attenton iteraction studied through event-related potentials. *J. Cogn. Neurosci.* 13 1109–1128. 10.1162/089892901753294400 11784449

[B13] Carretié L. Mercado F. Tapia M. Hinojosa J. A. (2001b). Emotion, attention, and the “negativity bias”, studied through event-related potentials. *Int. J. Psychophysiol.* 41 75–85. 10.1016/S0167-8760(00)00195-1 11239699

[B14] De Cesarei A. Peverato I. A. Mastria S. Codispoti M. (2015). Modulation of early ERPs by accurate categorization of objects in scenes. *J. Vis.* 15 1–14. 10.1167/15.8.14 26114677

[B15] Chib V. S. Rangel A. Shimojo S. O’Doherty J. P. (2009). Evidence for a common representation of decision values for dissimilar goods in human ventromedial prefrontal cortex. *J. Neurosci.* 29 12315–12320. 10.1523/JNEUROSCI.2575-09.2009 19793990 PMC6666137

[B16] Corbin J. C. Reyna V. F. Weldon R. B. Brainerd C. J. (2015). How reasoning, judgment, and decision making are colored by gist-based intutition: a fuzz-trace theory approach. *J. Appl. Res. Mem. Cognit.* 4 344–355. 10.1016/j.jarmac.2015.09.001 26664820 PMC4671075

[B17] Critchley H. Rolls E. T. (1996). Hunger and satiety modify the responses of the olfactory and visual neurons in the primate orbitofrontal cortex. *J. Neurophysiol.* 75 1673–1686. 10.1152/jn.1996.75.4.1673 8727405

[B18] Daw N. D. Niv Y. Dayan P. (2005). Uncertainty-based competition between prefrontal and dorsolateral striatal systems for behavioral control. *Nat. Neurosci.* 8 1704–1711. 10.1038/nn1560 16286932

[B19] De Martino B. Kumaran D. Holt B. Dolan R. J. (2009). The neurobiology of reference-dependent value computation. *J. Neurosci.* 29 3833–3842. 10.1523/JNEUROSCI.4832-08.2009 19321780 PMC2722101

[B20] Debener S. Thorne J. Schneider T. R. Campos Viola F. (2010). Using ICA for the analysis of multi-channel EEG data. *Oxford Scholar.* 6 45–66. 10.1093/acprof:oso/9780195372731.001.0001

[B21] Della Libera C. Chelazzi L. (2009). Learning to attend and to ignore is a matter of gains and losses. *Psychol. Sci.* 20 778–784. 10.1111/j.1467-9280.2009.02360.x 19422618

[B22] Delorme A. Makeig S. (2004). EEGLAB: an open source toolbox for analysis of single- trial EEG dynamics including independent component analysis. *J. Neurosci. Methods* 134 9–21. 10.1016/j.jneumeth.2003.10.009 15102499

[B23] Deppe M. Schwindt W. Kugel H. Plassman H. Kenning P. (2005). Nonlinear responses within the medial prefrontal cortex reveal when specific implicit information influences economic decision making. *J. Neuroimag.* 15 171–182. 10.1111/j.1552-6569.2005.tb00303.x 15746230

[B24] Dickinson A. Balleine B. (2002). “The role of learning in the operation of motivational systems” in *Steven’s Handbook of Experimental Psychology: Learning Motivation and Emotion*, eds Pashler H. Gallistel R. (Hoboken, NJ: John Wiley & Sons Inc.), 497–533.

[B25] Dimigen O. Sommer W. Hohlfeld A. Jacobs A. M. Kliegl R. (2011). Coregistration of eye movements and EEG in natural reading: analyses and review. *J. Exp. Psychol. Gen.* 140 552–572. 10.1037/a0023885 21744985

[B26] Furl N. Averbeck B. B. (2011). Parietal cortex and insula relate to evidence seeking relevant to reward-related decisions. *J. Neurosci.* 31 17572–17582. 10.1523/JNEUROSCI.4236-11.2011 22131418 PMC3474936

[B27] Gajewski P. D. Drizinsky J. Zülch J. Falkenstein M. (2016). ERP correlates of simulated purchase decisions. *Front. Neurosci.* 10:360. 10.3389/fnins.2016.00360 27551258 PMC4976093

[B28] Glimcher P. W. Fehr E. (2014). “Introduction: a brief history of neuroeconomics,” in *Neuroeconomics: Decision Making and the Brain* 2nd Edn, eds Glimcher P. W. Fehr E. (Oxford: Elsevier), 1–557.

[B29] Gluth S. Rieskamp J. Buchel C. (2012). Deciding when to decide: time-variant sequential sampling models explain the emergence of value-based decisions in the human brain. *J. Neuroisci.* 32 10686–10698. 10.1523/JNEUROSCI.0727-12.2012 22855817 PMC6621398

[B30] Goto N. Mushtaq F. Shee D. Lim X. L. Mortazavi M. Watabe M. (2017). Neural signals of selective attention are modulated by subjective preferences and buying decisions in a virtual shopping task. *Biol. Psychol.* 128 11–20. 10.1016/j.biopsycho.2017.06.004 28666891

[B31] Gottlieb J. (2007). From thought to action: the parietal cortex as a bridge between perception, action, and cognition. *Neuron* 53 9–16. 10.1016/j.neuron.2006.12.00 17196526

[B32] Gramann K. Gwin J. T. Bigdely-Shamlo N. Ferris D. P. Makeig S. (2010). Visual evoked responses during standing and walking. *Front. Hum. Neurosci.* 4:202. 10.3389/fnhum.2010.00202 21267424 PMC3024562

[B33] Gramann K. Jung T.-P. Ferris D. P. Lin C.-T. Makeig S. (2014). Toward a new cognitive neuroscience: modeling natural brain dynamics. *Front. Hum. Neurosci.* 8:444. 10.3389/fnhum.2014.00444 24994978 PMC4063167

[B34] Grech R. Cassar T. Muscat J. Camilleri K. P. Fabri S. G. Zervakis M. (2008). Review on solving the inverse problem in EEG source analysis. *J. Neuroeng. Rehabil.* 33 1–33. 10.1186/1743-0003-5-25 18990257 PMC2605581

[B35] Gwin J. T. Gramann K. Makeig S. Ferris D. P. (2010). Removal of movement artifact from high-density EEG recorded during walking and running. *J. Neurophysiol.* 103 3526–3534. 10.1152/jn.00105.2010 20410364 PMC3774587

[B36] Hanatani T. Sumi N. Taguchi S. Fugimoto O. Nan-no H. Takeda M. (2005). Event-related potentials in panic disorder and generalized anxiety disorder. *Psychiatry Clin. Neurosci.* 59 83–88. 10.1111/j.1440-1819.2005.01336.x 15679545

[B37] Handy T. C. Smilek D. Geiger L. Liu C. Schooler J. W. (2010). ERP evidence for rapid hedonic evaluation of logos. *J. Cognit. Neurosci.* 22 124–138. 10.1162/jocn.2008.21180 19199410

[B38] Hoechstetter K. Berg P. Scherg M. (2010). *BESA Research Tutorial 4: Distributed Source Imaging*, 1–29. Available at: ftp://www.besa.de/be/besa.de/demonstrations_and_tutorials/BESA%20Reserach%20Tutorial%204%20-%20Distributed%20Source%20Imaging.pdf

[B39] Hsu C.-W. Goh J. O. S. (2016). Distinct and overlapping brain areas engaged during value-based, mathematical, and emotional decision processing. *Front. Hum. Neurosci.* 10:275. 10.3389/fnhum.2016.00275 27375466 PMC4901075

[B40] Huang Y. Luo Y. (2006). Temporal course of emotional negativity bias: an ERP study. *Neurosci. Lett.* 398 91–96. 10.1016/j.neulet.2005.12.074 16446031

[B41] Hubert M. Kenning P. (2008). A current overview of consumer neuroscience. *J. Consum. Behav.* 7 272–292. 10.1002/cb.251 25970630

[B42] IBM Corp (2016). *IBM SPSS Statistics for Windows, Version 24.0.* Armonk, NY: IBM Corp.

[B43] Iriarte J. Urrestarazu E. Valencia M. Alegre M. Malanda A. Viteri C. (2003). Independent component analysis as a tool to eliminate artifacts in EEG: a quantitative study. *J. Clin. Neurophysiol.* 20 249–257. 10.1097/00004691-200307000-00004 14530738

[B44] Jones W. J. Childers T. L. Jiang Y. (2012). The shopping brain: math anxiety modulates brain responses to buying decisions. *Biol. Psychol.* 89 201–213. 10.1016/j.biopsycho.2011.10.011 22027087

[B45] Jung T. Makeig S. Humphries C. Lee T. McKeown M. J. Iragui I. (2000). Removing electroencephalographic artefacts by blind source seperation. *Psychophysiology* 37 163–178. 10.1111/1469-8986.3720163 10731767

[B46] Jungnickel E. Gramann K. (2016). Mobile brain/body imaging (MoBI) of physical interaction with dynamically moving objects. *Front. Hum. Neurosci.* 10:306. 10.3389/fnhum.2016.00306 27445747 PMC4921999

[B47] Kahneman D. Tversky A. (1979). Prospect theory: An analysis of decision under risk. *Econometrica* 47 263–292. 10.2307/1914185

[B48] Keller L. R. Segal U. Wang T. (1993). The becker-degroot-marschak mechanism and generalized utility theories: theoretical predictions and empirical observations. *Theory Decis.* 34 83–97. 10.1007/BF01074895

[B49] Khushaba R. N. Wise C. Kodagoda S. Louviere J. Kahn B. E. Townsend C. (2013). Consumer neuroscience: assessing the brain response to marketing stimuli using electroencephalogram (EEG) and eye tracking. *Expert. Syst. Appl.* 40 3803–3812. 10.1016/j.eswa.2012.12.095

[B50] Kirchner H. Thorpe S. J. (2006). Ultra-rapid object detection with saccadic eye movements: visual processing speed revisited. *Vis. Res.* 46 1762–1776. 10.1016/j.visres.2005.10.002 16289663

[B51] Krawczyk D. C. Gazzaley A. D’Esposito M. (2007). Reward modulation of prefrontal and visual association cortex during an incentive working memory task. *Brain Res.* 1141 168–177. 10.1016/j.brainres.2007.01.052 17320835

[B52] Ladouce S. Donaldson D. I. Dudchenko P. A. Ietswaart M. (2017). Understanding minds in real-world environments: toward a mobile cognition approach. *Front. Hum. Neurosci.* 10:694. 10.3389/fnhum.2016.00694 28127283 PMC5226959

[B53] Lau T. M. Gwin J. T. Ferris D. P. (2012). How many electrodes are really needed for EEG-based mobile brain imaging?. *Behavi. Brain Sci.* 2 387–393. 10.4236/jbbs.2012.23044

[B54] Lehmann D. (1987). “Principles of spatial analysis,” in *Handbook of Electroencephalography and Clinical Neuropsychology, Rev. Ser. Vol. 1: Methods of Analysis of Brain Electrical and Magnetic Signals*, eds Gevins A. S. Redmond A. (Amsterdam: Elsevier), 209–354.

[B55] Lin A. Adolphs R. Rangel A. (2012). Social and monetary reward learning engage overlapping neural substrates. *Soc. Cogn. Affect. Neurosci.* 7 274–281. 10.1093/scan/nsr006 21427193 PMC3304477

[B56] Luck S. J. (2005). *An Introduction to the Event-Related Potential Technique.* London: the MIT Press.

[B57] Mahesan D. Chawla M. Miyapuram K. P. (2016). “The effect of reward information on perceptual decision-making,” in *Neural Information Processing, ICONIP. Lecture Notes in Computer Science*, eds Hirose A. Ozawa S. Doya K. Ikeda K. Lee M. Liu D. (Cham: Springer).

[B58] Maris E. Oostenveld R. (2007). Nonparametric statistical testing of EEG- and MEG-data. *J. Neurosci. Methods* 164 177–190. 10.1016/j.jneumeth.2007.03.024 17517438

[B59] Menon V. Rivera S. M. White C. D. Glover G. H. Reiss A. L. (2000). Dissociating prefrontal and parietal cortex activation during arithmetic processing. *NeuroImage* 12 357–365. 10.1006/nimg.2000.0613 10988030

[B60] Minati L. Grisoli M. Franceschetti S. Epifani F. Granvillano A. Medford N. (2012). Neural signatures of economic parameters during decision-making: a functional MRI (fMRI), electroencephalography (EEG) and autonomic monitoring study. *Brain Topogr.* 25 73–96. 10.1007/s10548-011-0210-1 22101380

[B61] Minguillon J. Lopez-Gordo M. A. Pelayo F. (2017). Trends in EEG-BCI for daily-life: requirements for artifact removal. *Biomed. Signal Process. Control* 31 407–418. 10.1016/j.bspc.2016.09.005

[B62] Morris R. W. Dezfouli A. Griffiths K. R. Balleine B. W. (2014). Action-value comparisons in the dorsolateral prefrontal cortex control choice between goal-directed actions. *Nat. Commun.* 5 1–10. 10.1038/ncomms5390 25055179 PMC4124863

[B63] Nakatani C. Chehelcheraghi M. Jarrahi B. Hironori N. Leeuwen C. V. (2013). Cross-frequency phase synchrony around the saccade period as a correlate of perceiver’s internal state. *Front. Syst. Neurosci.* 7:18 10.3389/fnsys.2013.00018 PMC3664768 23754990

[B64] Nee D. E. Wager T. D. Jonides J. (2007). Interference resolution: insights from a meta- analysis of neuroimaging tasks. *Cogn. Affect. Behav. Neurosci.* 7 1–17. 10.3758/CABN.7.1.1 17598730

[B65] Nikolaev A. R. Meghanathan R. N. van Leeuwen C. (2016). Combining EEG and eye movement recording in free viewing: pitfalls and possibilities. *Brain Cogn.* 107 55–83. 10.1016/j.bandc.2016.06.004 27367862

[B66] Padoa-Schioppa C. Assad J. A. (2006). Neurones in the orbitofrontal cortex encode economic value. *Nature* 441 223–226. 10.1038/nature04676 16633341 PMC2630027

[B67] Palmer J. A. Makeig S. Kreutz-Delgado K. Rao B.D. (2008). “Newton method for the ICA mixture model,” in *Proceedings of the 33rd IEEE International Conference on Acoustics and Signal Processing*, Las Vagas, NV 1805–1808.

[B68] Pessoa L. Engelmann J. B. (2010). Embedding reward signals into perception and cognition. *Front. Neurosci.* 4:17. 10.3389/fnins.2010.00017 20859524 PMC2940450

[B69] Peters J. Büchel C. (2010). Neural representations of subjective reward value. *Behav. Brain Res.* 213 135–141. 10.1016/j.bbr.2010.04.031 20420859

[B70] Pexman P. M. Hargreaves I. S. Edwards J. D. Henry L. C. Goodyear B. G. (2007). The neural consequences of semantic richness: when more comes to mind, less activation is observed. *Psychol. Sci.* 18 401–407. 10.1111/j.1467-9280.2007.01913.x 17576279

[B71] Picton T. Bentin S. Berg P. (2000). Guidelines for using human event-related potentials to study cognition: recording standards and publication criteria. *Psychophysiology* 37 127–152. 10.1111/1469-8986.3720127 10731765

[B72] Plassmann H. O’Doherty J. Rangel A. (2007). Orbitofrontal cortex encodes willingness to pay in everyday economic transactions. *J. Neurosci.* 27 9984–9988. 10.1523/JNEUROSCI.2131-07.2007 17855612 PMC6672655

[B73] Plassmann H. O’Doherty J. P. Rangel A. (2010). Appetitive and aversive goal values are encoded in the medial orbitofrontal cortex at the time of decision making. *J. Neurosci.?* 30 10799–10808. 10.1523/JNEUROSCI.0788-10.2010 20702709 PMC6634706

[B74] Polanía R. Krajbich I. Grueschow M. Ruff C. C. (2014). Neural oscillations and synchronization differentially support evidence accumulation in perceptual and value-based decision making. *Neuron* 82 709–720. 10.1016/j.neuron.2014.03.014 24811387

[B75] Polezzi D. Lotto L. Daum I. Sartori G. Rumiati R. (2008). Predicting outcomes of decisions in the brain. *Behav. Brain Res.* 187 116–122. 10.1016/j.bbr.2007.09.001 17935798

[B76] Pozharliev R. Verbeke W. J. M. I. Van Strien J. W. Bagozzi R. P. (2015). Merely being with you increases my attention to luxury products: using EEG to understand consumers’, emotional experience with luxury branded products. *J. Mark. Res.* 52 546–558. 10.1509/jmr.13.0560

[B77] Pradeep A. K. (2010). *The Buying Brain: Secrets for Selling to the Subconscious Mind.* New York, NY: John Wiley & Sons.

[B78] Rangel A. Camerer C. Montague P. R. (2008). A framework for studying the neurobiology of value-based decision making. *Nat. Rev. Neurosci.* 9 545–556. 10.1038/nrn2357 18545266 PMC4332708

[B79] Reis P. Hebenstreit F. Gabsteiger F. von Tscharner V. Lochmann M. (2014). Methodological aspects of EEG and body dynamics measurements during motion. *Front. Hum. Neurosci.* 8:156. 10.3389/fnhum.2014.00156 24715858 PMC3970018

[B80] Ruff C. C. Fehr E. (2014). The neurobiology of rewards and values in social decision making. *Nat. Rev. Neurosci.* 15 549–562. 10.1038/nrn3776 24986556

[B81] Samuelson P. (1937). Note on measurement of utility. *Rev. Econ. Stud.* 4 155–161. 10.2307/2967612

[B82] San Martín R. (2012). Event-related potential studies of outcome processing and feedback-guided learning. *Front. Hum. Neurosci.* 6:304. 10.3389/fnhum.2012.00304 23162451 PMC3491353

[B83] Scherg M. Von Cramon D. (1986). Evoked dipole source potentials of the human auditory cortex. *Electroencephalogr. Clin. Neurophysiol.* 65 344–360. 10.1016/0168-5597(86)90014-6 2427326

[B84] Schienle A. Scharmuller W. Schwab D. (2017). Processing of visual food cues during bitter taste perception in female patients with binge-eating symptoms?: a cross-modal ERP study. *Clin. Neurophysiol.* 128 2184–2190. 10.1016/j.clinph.2017.08.017 28950151

[B85] Schultz W. Apicella P. Ljungberg T. (1993). Responses of monkey dopamine neurons to reward and conditioned stimuli during successive steps of learning a delayed response task. *J. Neurosci.* 13 900–913. 10.1523/JNEUROSCI.13-03-00900.1993 8441015 PMC6576600

[B86] Schupp H. T. Cuthbert B. N. Bradley M. M. Cacioppo J. T. Ito T. Lang P. J. (2000). Affective picture processing: the late positive potential is modulated by motivacional relevance. *Psychophysiology* 37 257–261. 10.1111/1469-8986.3720257 10731776

[B87] Seghier M. L. (2013). The angular gyrus: multiple functions and multiple subdivisions. *Neuroscientist* 19 43–61. 10.1177/1073858412440596 22547530 PMC4107834

[B88] Soto V. Tyson-Carr J. Kokmotou K. Roberts H. Cook S. Fallon T. (2018). Brain responses to emotional faces in natural settings: A wireless mobile EEG recording study. *Front. Psychol.* 9:2003. 10.3389/fpsyg.2018.02003 30410458 PMC6209651

[B89] Stancak A. Hoechstetter K. Tintera J. Vrana J. Rachmanova R. Kralik J. (2002). Source activity in the human secondary somatosensory cortex depends on the size of corpus callosum. *Brain Res.* 936 47–57. 10.1016/S0006-8993(02)02502-7 11988229

[B90] Stott J. J. Redish A. D. (2015). Representations of value in the brain: an embarrassment of riches? *PLoS Biol.* 13:e1002174. 10.1371/journal.pbio.1002174 26086790 PMC4472787

[B91] Swirski L. Bulling A. Dodgson N. (2012). Robust real-time pupil tracking in highly off- axis images. *Etra* 1 173–176. 10.1145/2168556.2168585

[B92] Telpaz A. Webb R. Levy D. J. (2015). Using EEG to predict consumers’ future choices. *J. Mark. Res.* 52 511–529. 10.1509/jmr.13.0564

[B93] Thickbroom G. W. Knezeviè W. Carroll W. M. Mastaglia F. L. (1991). Saccade onset and offset lambda waves: relation to pattern movement visually evoked potentials. *Brain Res.* 551 150–156. 10.1016/0006-8993(91)90927-N 1913148

[B94] Thickbroom G. W. Mastaglia F. L. (1986). Presaccadic spike potential. Relation to eye movement direction. *Electroencephalogr. Clin. Neurophysiol.* 64 211–214. 10.1016/0013-4694(86)90167-7 2427314

[B95] Tyson-Carr J. Kokmotou K. Soto V. Cook S. Fallon N. Giesbrecht T. (2018). Neural correlates of economic value and valuation context. *J. Neurophysiol.* 119 1924–1933. 10.1152/jn.00524.2017 29442556

[B96] Wallis J. D. Rich E. L. (2011). Challenges of interpreting frontal neurons during value- based decision-making. *Front. Neurosci.* 5:124. 10.3389/fnins.2011.00124 22125508 PMC3222102

[B97] Wilkinson N. Klaes M. (2012). *An Introduction to Behavioral Economics.* Basingstoke: Palgrave Macmillan 10.1007/978-0-230-39103-1

[B98] Xie J. Padoa-Schioppa C. (2016). Neuronal remapping and circuit persistence in economic decisions. *Nat. Neurosci.* 19 855–863. 10.1038/nn.4300 27159800 PMC4882218

[B99] Yagi A. (1979). Lambda waves associated with offset of saccades: a subject with large lambda waves. *Biol. Psychol.* 8 235–238. 10.1016/0301-0511(79)90051-6 497316

[B100] Yagi A. (1981). Visual signal detection and lambda responses. *Electroencephalogr. Clin. Neurophysiol.* 52 604–610. 10.1016/0013-4694(81)91434-6 6172259

[B101] Yeung N. Sanfey A. G. (2004). Independent coding of reward magnitude and valence in the human brain. *J. Neurosci.* 24 6258–6264. 10.1523/JNEUROSCI.4537-03.2004 15254080 PMC6729539

